# A Novel Virtual Coaching System Based on Personalized Clinical Pathways for Rehabilitation of Older Adults—Requirements and Implementation Plan of the vCare Project

**DOI:** 10.3389/fdgth.2020.546562

**Published:** 2020-10-05

**Authors:** Sofoklis Kyriazakos, Hannes Schlieter, Kai Gand, Massimo Caprino, Massimo Corbo, Peppino Tropea, Elda Judica, Irma Sterpi, Stefan Busnatu, Patrick Philipp, Jordi Rovira, Alvaro Martínez, Marc Lange, Inigo Gabilondo, Rocio Del Pino, Juan Carlos Gomez-Esteban, Lucia Pannese, Morten Bøttcher, Vibeke Lynggaard

**Affiliations:** ^1^Department of Business Development and Technology, Aarhus University, Aarhus, Denmark; ^2^Faculty of Business and Economics, Technische Universität Dresden, Dresden, Germany; ^3^Department of Neurorehabilitation Sciences, Casa di Cura del Policlinico, Milano, Italy; ^4^Universitatea de Medicina si Farmacie “Carol Davila” din Bucuresti, Bucuresti, Romania; ^5^FZI Forschungszentrum Informatik am Karlsruher Institut für Technologie, Karlsruhe, Germany; ^6^MYSPHERA, Valencia, Spain; ^7^European Health Telematics Association, Brussels, Belgium; ^8^Biocruces-Bizkaia Health Research Institute, Bilbao, Spain; ^9^Imaginary s.r.l., Milan, Italy; ^10^Hospitalsenheden Vest, Herning, Denmark

**Keywords:** virtual coaching, personalized medicine, clinical pathways (CPs), home rehabilitation, digital health (eHealth), embodied conversational agents

## Abstract

Home-based rehabilitation after an acute episode or following an exacerbation of a chronic disease is often problematic with a clear lack of continuity of care between hospital and home care. Secondary prevention is an essential element of long-term rehabilitation where strategies oriented toward risk reduction, treatment adherence, and optimization of quality of life need to be applied. Frail and sometimes isolated, the patient fails to adhere to the proposed post-discharge clinical pathway due to lack of appropriate clinical, emotional, and informational support. Providing a suitable rehabilitation after an acute episode or a chronic disease is a major issue, as it helps people to live independently and enhance their quality of life. However, as the rehabilitation period usually lasts some months, the continuity of care is often interrupted in the transition from hospital to home. Virtual coaches could help these patients to engage in a personalized rehabilitation program that complies with age-related conditions. These coaches could be a key technology for empowering patients toward increasing their adherence to the care plan and to improve their secondary prevention measures. In this paper, we are presenting a novel virtual coaching system that will address these challenges by combining recent technological advances with clinical pathways, based on joint research and validation activities from researchers from the medical and information and communication technology (ICT) domains.

## Introduction

Non-communicable diseases (NCDs) represent the leading public health challenges globally in the 21st century, resulting in ill health, economic burden, life loss, diminished quality of life (QoL), and poor social development equally in both high-resourced and low-resourced countries. NCDs include mostly strokes, heart diseases, Parkinson's disease (PD), cancers, diabetes, chronic kidney disease, respiratory diseases, and Alzheimer's disease. We have got to know to a point which one out of six people in the EU has a disability (1) caused by a chronic evolving disease or an acute episode. In addition, having a life with disabilities, given the inappropriate management of the disease risk factors according to the World Health Organization (WHO) reports, globally, NCD deaths will increase by 17% in the following 10 years, with the most considerable increase being in low-resourced countries such as in Africa (27%) and the Eastern Mediterranean region (25%) ^1^.

That is why all health-care organizations strive to improve their strategy for disease prevention and control in order to maintain the health of the community. The efficient implementation of the secondary prevention measures through adequate rehabilitation programs represents a primary issue for patients with NCD as they age, helping them to enhance their QoL and to live independently. However, given that the institutionalized and telemonitored rehabilitation programs usually last for a standardized period, due to the reduced number of caregivers associated with the increased costs for the service, the continuity of care is often interrupted in the transition from the institutionalized rehabilitation to the home self-rehabilitation environment. Using today's intelligent monitoring and interaction technologies, virtual coaches (VCs) could support these patients to continue with a personalized rehabilitation at home, adjusted to the age-related conditions. This way, also, they partly compensate the absence of a human caregiver. By natural language processing interaction, facial recognition, and behavioral monitoring, VCs could motivate patients to engage and perform their recommended physical activity routine, to stick to the medication plan, to avoid unhealthy habits, and to keep their social life active. In sum, such VCs could be key measures for the empowerment of patients toward implementing long-life healthy actions.

### User Needs in Virtual Coaching-Based Rehabilitation Scenarios

Home rehabilitation intends to re-build the patients independence and to reduce their psychological stress, so that they can take part again in different daily activities or even to re-enter the economic circuit and to become a productive member of the society (2). In particular, patients with non-communicable chronic diseases such as PD, ischemic heart disease (IHD), and heart failure (HF) need a continuing care process. Usually, a specialized team of physicians and carers is necessary to support the patient to adhere to the care plans throughout life in order to avoid exacerbation of the disease or the appearance of other comorbidities. Although the medical caregivers define a complete treatment schema composed of medication, nutrition, and physical activity recommendations, it is still quite hard for the patient to transfer those recommendations fully to daily routines. As depicted in **Figure 4**, the approach of a VC is to accompany the professional work with practical tool that engages patients to work on their risk factors and motivates them into implementing the healthy measures.

Home VC-assisted rehabilitation could be indicated when the patient is able to engage in several suggested activities and when learning and skill development is required for long-period therapy continuation. Meaningful planning and responsiveness are the key determinants of home rehabilitation for improving the QoL of the patient^2^. Furthermore, due to the medical caregivers' reduced presence, home rehabilitation could be less costly as compared with the care endured in the hospital under a professional's observation (3). Overall, patients' needs (for virtual coaching scenarios) for home rehabilitation include

- **Physical activity**—Physical activity for exercises of varying intensity to revive motor function, health screening with vital signs measurement, and adjustment for the next exercise session in a real-time situation^3^. Instructed by a VC, the patient develops the feeling of safety as the activity is monitored by a system that follows the treatment plan as set up by the patient's medical professional.
- **Vital signs or parameters monitoring**—Assessment of parameters when there is no physical activity to see whether the medical therapy is acting as expected.
- **Smart behavioral pattern analysis**—Using Internet of Things (IoT) solutions for the seamless collection of different sources of data (e.g., patient location, environment, and health) and analysis.
- **Compensatory strategies**—Especially in the event that a care plan is not executed properly, allowing the system to give feedback. The user needs to be continuously engaged until he/she becomes habituated to the activities suggested (4).
- **User education and therapy effectiveness**—Counseling, to make him/her aware of the disease severity and consequences of non-adherence. It is essential for a user and associated family members to be actively involved in the care plan as the success of the rehabilitation depends on their active engagement.
- **Behavioral modification/motivational or psychological strategies**—It is essential for the patient to adhere to the care plan through constant reminders or through strategies to develop healthy habits. An intelligent decision-making approach is needed to change behavioral patterns complying to the needs of the user.
- **Assistive technological measures**—Easy-to-use and easily accessible assistive technological measures, e.g., a tablet application, an avatar, or a VC. Serious games (as a mean of home physical activity option) should be level- or intensity-adjustable based on patients' clinical evolution and based on his/her likeliness. These games should be motivational to avoid loss of interest.
- **User literacy**—Education of patients regarding disease, treatment, or handling of a device. It should be the objective of any rehabilitation strategy to encourage the patient to be independent.
- **Device portability**—Any device or sensor that is attached should be lightweight and comfortable and should be focused on the body part involved.

User needs are aligned with the input data analysis results, as mentioned above. For the success of a planned care rehabilitation, at home and not having permanent assistance from medical staff is a key aspect. Thus, a user-friendly coaching system ensuring guidance, assessment with immediate feedback strategy, and continuous engagement is needed. For ensuring patients' appropriate therapy, the coach should act personally and should provide assistance as required by the users' needs and individual rehabilitation care pathways. This will be outlined in vCare's virtual coaching concept.

### Related Work

Self-management home-based rehabilitation solutions are often lacking specialized equipment and have insufficient alignment with patient's needs. Adaptability of the technical means to the individual care needs and abilities is mostly not provided. In the literature, several studies have analyzed the interaction between virtual coaching and users in various clinical settings and for different purposes. We have focused our selection on those studies that are dominant and throughout the years remain reference points in this field.

Ding et al. (5) highlighted that one of the greatest areas of innovation for virtual coaching was to support preventative health management and self-care. They believed that a new class of intelligent devices and applications could have facilitated self-management, users' compliance, and prevention of secondary conditions in the field of rehabilitation. In (6), a sustained level of activity in overweight adults provided with a rule-based virtual coaching system plus a pedometer and web-based feedback, in comparison with a decline seen in those without virtual coaching, was demonstrated. Admittedly, the use of a rule-based e-coach support helped the users to keep their weight under control (7, 8). Further, the development of a rule-based embodied conversational agent (ECA) persuaded people to improve their eating habits (9). Thus, first, results can be observed here, but the research seems to be still in a very early phase with regard to the technological fitness and clinical evidence.

In (10), VCs and comfortable human–computer interfaces, based on user-based artificial intelligence (AI) measures, promoted active information processing and adoption with regard to motivation and behavior changes. The swift communication was a particularly important aspect of this. However, high-quality e-health communication programs also rely on appropriate and dynamic messages exchanged than only on the digital channels used.

Interestingly, in a single case presented in (11), a powerlifter under long-distance coaching by the cardiac rehabilitation staff managed to return to the professional sport level after coronary artery bypass grafting. By making use of high-intensity training plus phone and email support, he was able to lift heavier loads than he had before the heart surgery. Concerning a further cardiologic disease, Ritchie et al. (12) studied the use of the system and readmissions of a pragmatic randomized trial of an e-coach in a sample of adults with chronic HF (CHF) and chronic obstructive pulmonary disease (COPD) from the southern USA. Although 30-day re-hospitalization rates did not significantly differ between the e-coach and usual post-discharge care groups, in the COPD subgroup, the use of the e-coach led to significantly fewer days in the hospital. This indicates that such interventions should be disease specific to enhance the effectiveness in lowering re-hospitalization rates and improving post-discharge care.

Virtual coaching can furthermore be an efficient and low-cost approach offering an innovative opportunity to improve patient/clinician partnerships in managing chronic conditions, in particular in the improvement of patient/clinician communication (3).

For the elder population, Bickmore et al. (13) and (14) evaluated the efficacy of an automated intervention with an ECA to test improvements of physical activity and fruit and vegetable intake. These studies showed promising results at changing health behavior. Other authors (15) used ECA for engaging elder people in regular physical exercise. Results showed a substantial potential to decrease the health disparity gap by promoting key health behaviors in underserved populations. Additionally, it has been studied how the older adults could interact with a pet avatar: a pilot study (16) was conducted to examine the perceived acceptance and utility of a tablet-based conversational agent in the form of a pet avatar used by older adults during daily interactions over 3 months. It could have been shown that this pet interface provides older adults with worthwhile companionship, enhancing social interaction. Recently, also the possible application of ECAs in clinical psychology has been reviewed. However, the result shows only a limited applicability by now (17).

Although the literature on virtual coaching still reports a limited number of subjects involved and few clinical conditions investigated, by using rule-based coaching systems, data on the use of internet-based coaching are encouraging and show that people can get used to electronic communication in order to take advantage of it for their state of health; a recent scoping review on virtual coaching (18) concludes that, in this scenario, new studies on these innovative and intelligent solutions are necessary. In particular, for patients with disabilities due to chronic neurological and cardiologic conditions, the virtual coaching system could be a preferred solution for conducting personalized rehabilitation programs at home, which can guarantee continuity of care outside hospital premises.

### Related Projects

Further to the scientific literature, several research projects (apart from, but partly complementary to vCare) integrate technology of virtual coaching, such as the following: Well-being and Health Virtual Coach (WellCO) is a VC that is intended to cater to seven areas of human health, namely, cognitive stimulation, leisure and entertainment, support groups, physical activity, health status, nutrition, and tips (11). These areas pertain to a whole range of age groups and reflect a person's well-being in any condition, whereas holograms for personalized virtual coaching and motivation in an aging population with balance disorders (HOLOBALANCE) is a personalized coaching solution for people with balance disorders mainly concerning age-related progressive loss of sensory information^4^. Empathic, Expressive, Advanced Virtual Coach to Improve Independent Healthy-Life-Years of the Elderly (EMPATHIC) is a VC to engage healthy senior users to take care of their lifestyle with a significant degree of independence^5^. The solutions help to take care of chronic diseases and follow a healthy diet with adequate physical activities by integrating the user with sufficient social functions. The Council of Coaches is another EU H2020 project that provides conversational agents that enable fluent multi-party interaction between multiple coaches and users, thereby changing the coaching paradigm^6^. Novel Empowering Solutions and Technologies for Older people to Retain Everyday life activities (NESTORE) is a behavioral-based iterative virtual coaching that plans the coaching schedules based on mood and environment^7^. NESTORE is an elderly-oriented solution that promotes healthy aging and is committed to user privacy. Coach Assistant via Projected and Tangible Interface or CAPTAIN is a human–computer interface with radical approaches, such as augmented reality, for non-invasive collection and analysis of emotional, behavioral, and physiological data, and has a motivational engine to guide users for healthy diet and exercise tips, cognitive activities, and social interactions^8^. Supporting Active Aging through Multimodal Coaching, as the name suggests, is a server- and client-sided platform for specialized well-being monitoring and assessment. The platform endorses ambient data collection requiring minimal user input with immense ease of use. Widening the support for large-scale uptake of Digital Innovation for Active and Healthy Aging (WE4AHA) shall build a thorough set of promotion and support services that will embellish the use of Digital Innovation for Active Healthy Aging (AHA)^9^. Through WE4HA, the relevant stakeholders will be able to develop and implement three EU-guided activities, namely, innovation to Market (I2M)^10^, Blueprint Digital Transformation of Health and Care for the Aging Society, and EIP on AHA.

The above-mentioned projects have been compared with vCare in terms of the following criteria: (a) secondary prevention approach, as secondary prevention using VC is usually neglected; (b) personalized care plan through adaptive care pathways in opposition with standardized care plan managed manually by clinician/caregiver instead of dynamic adaptation; (c) continuity of care, as it brings clinical focus to home contextualization, usually neglected in the clinical practice; (d) extended QoL perspective, as it focuses on multiple aspects of the secondary prevention other that rehabilitation; and (e) multiple clinical settings, taking charge of two different clinical contexts but for some related aspects due to severity and cure modalities, very close to each other.

In sum, the related work shows that there is significant need to investigate virtual coaching for rehabilitation cases. As there is less evidence on the design and validation of VC systems, we summarize the main design principles of the vCare concept in the following chapter.

### Paper Structure

The paper summarizes the conceptual work for a novel VC system based on personalized clinical pathways. It basically consolidates the finding of the project “vCare” (Virtual Coaching Activities for Rehabilitation in Elderly), which is funded by the European Commission to advance the field of virtual coaching activities. The ambition for building an intelligent VC is mainly due to the scarcity of evidence for particular VC-based treatments (18), the application of a participatory design, and the continuous personalization of the care pathways concerning changing habits, context information, and personal preferences of the patient. In the paper we show how the vCare project is addressing these ambitions. Besides the conceptual framing of our solution, we also outline the integration of different technologies such as monitoring and location devices, serious games, coaching services, human-like avatars as interfaces, and machine learning technology to realize the intelligent VC.

This paper is organized into six sections. Following the abstract and the introduction, in *Materials and Equipment*, we present the materials and equipment, focusing on the vCare approach and the underlying architecture. In *Methods*, we present the methodologies by listing the four pathologies that we are addressing, as well as the chosen validation framework. In *Anticipated Results*, we present the clinical endpoints with quantified key performance indicators. *Discussion* is about the conclusions and the future work.

## Materials and Equipment

### vCare Approach

vCare develops and investigates a new information and communication technology (ICT)-based concept, encapsulating a set of coaching services for empowering and motivating people, helping them to proceed with a personalized rehabilitation that complies with age-related physical, cognitive, mental, and social conditions by a VC.

**Figure 1:**
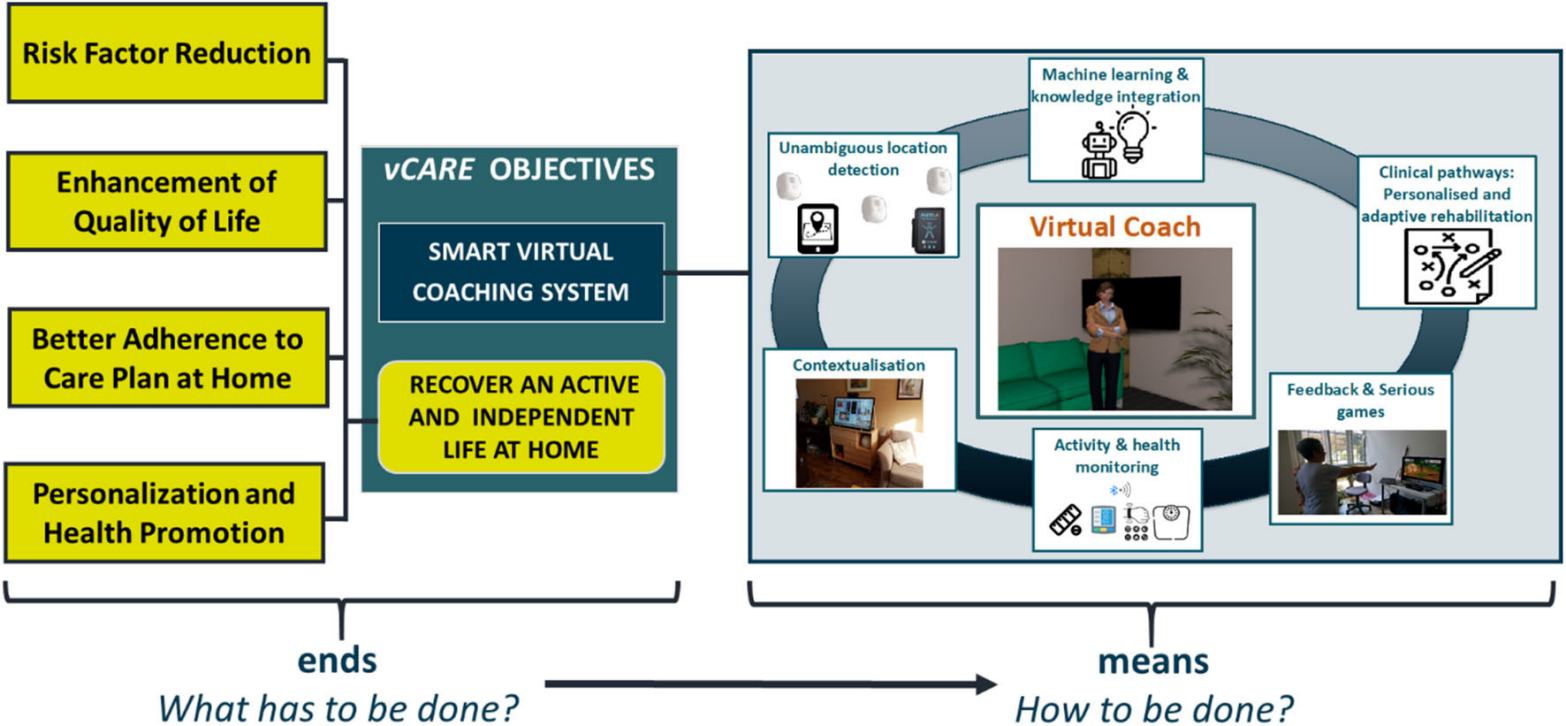
vCare's ends and means.

In particular, by utilizing the VC, we aim to enhance the QoL of patients by helping them to adhere to the care plan that consisted of personalized healthy recommendations for the daily activities. The coaching will lead to a continuous reduction of risk factors that are related to the probability of a relapse of the disease, the manifestation of disabilities, or the decline of (mental) health (see Figure 1).

vCare monitors the patients' daily activities, conditions, and habits to provide personalized feedback aiming to improve its QoL. For that, the coaching programs are initialized by specialists by assigning a well-elaborated clinical pathway as a tailored rehabilitation plan for the patient (in accordance with the personal context information and specific patients' needs). According to this plan, vCare enables the personalization of the intensity and way of interaction and will trigger suitable exercises (serious games, cognitive training programs, etc.), suggestions, and feedback. Additionally, the context information that helps to monitor daily life activities and individual habits will be integrated.

**Figure 2:**
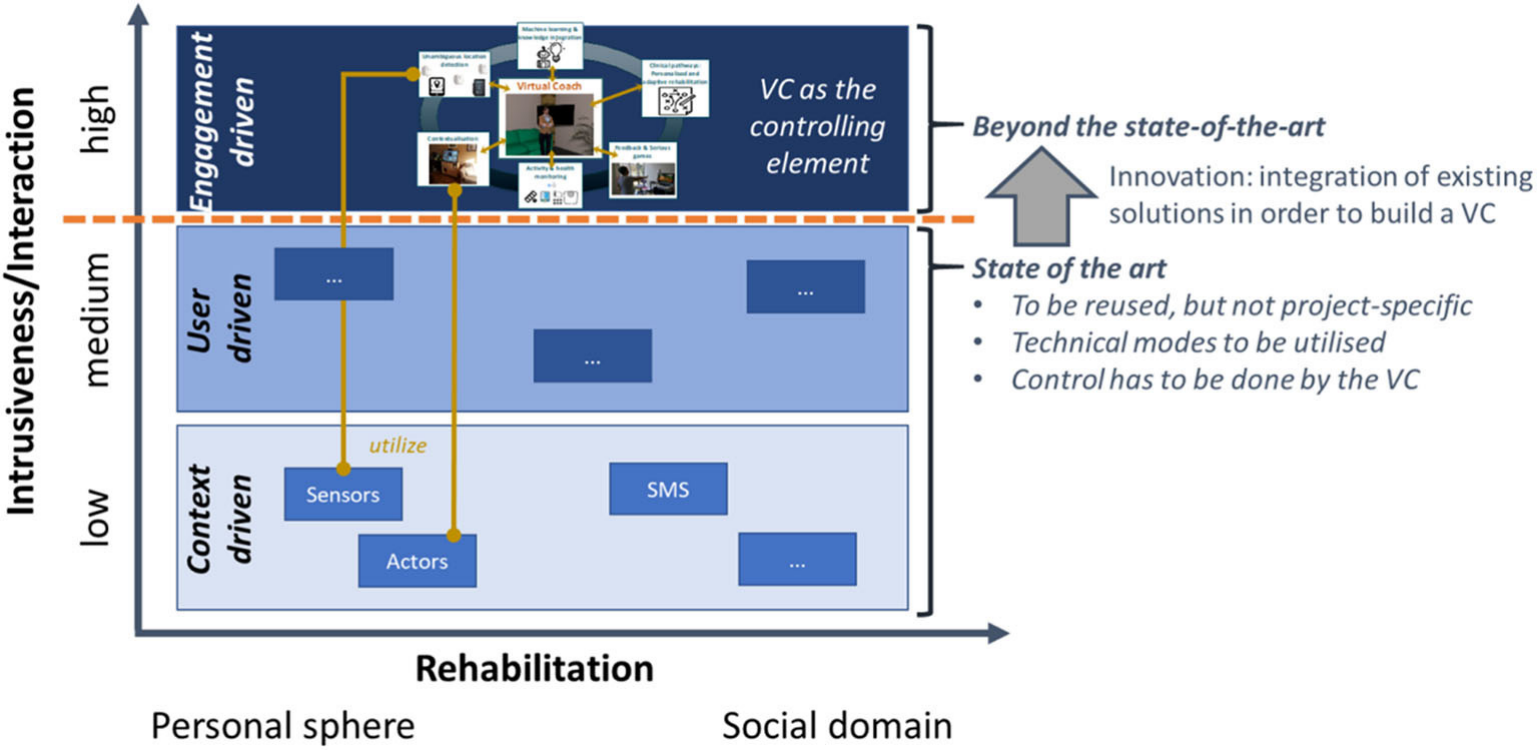
Meaning of “beyond the state-of-the-art” —unique selling point of vCare.

vCare integrates semantic technologies (reasoning, behavioral models, predictive analytics, and going beyond only rule-based systems but more intelligent adaptations), well-elaborated coaching services, and clinical pathway services having the VC as the central and controlling element (see Figure 2).

**Figure 3:**
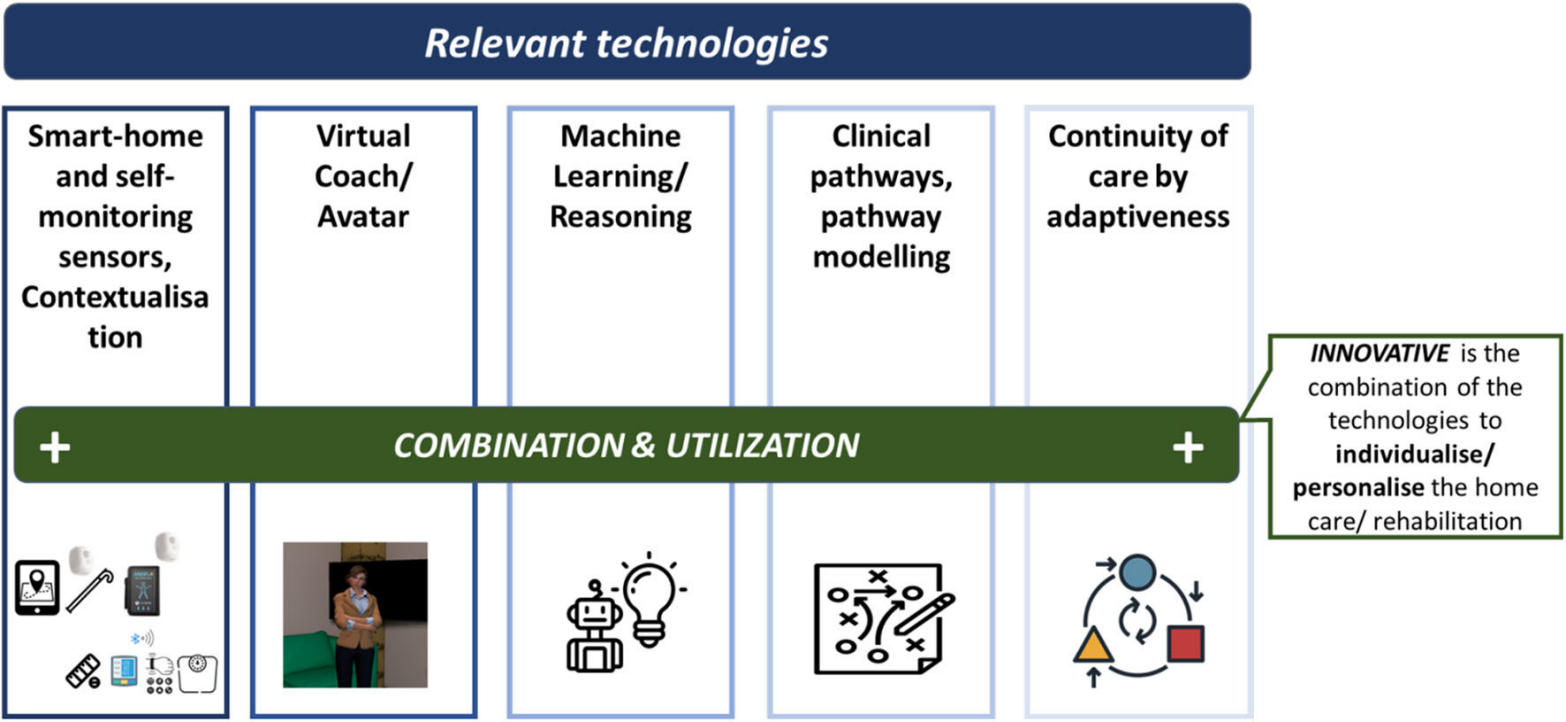
Combining different technologies as an innovative element of vCare.

vCare channels the interaction with the patient at home via a virtual avatar, which communicates mainly, but not exclusively, via natural speech communication. Alternative modalities depend on the users' preferences or context. The VC encapsulates coaching and supportive services as well telemonitoring services. The latter provide the data-related basis for the VC's decision logic and tracing the data that the service provision is based on. However, the innovativeness of vCare does not only reside in the utilization of single technologies but in the combination of these technologies (see Figure 3). This is to individualize/personalize the home care/rehabilitation and somehow fill the gap that the caregiver's absence has caused.

### Adaptiveness and Personalization by the Means of the Virtual Coach

**Figure 4:**
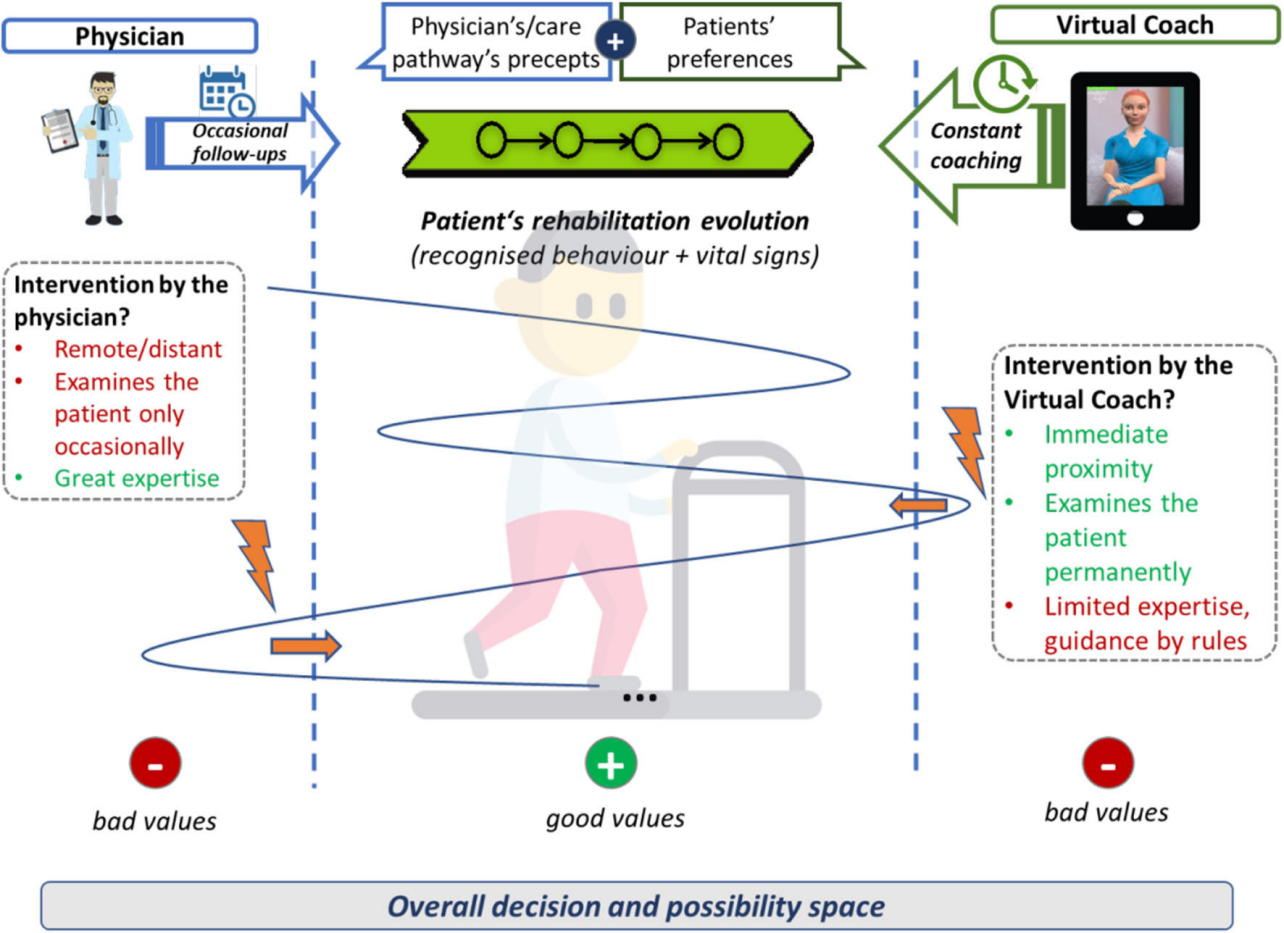
Decision and possibility space and respective possible interventions.

Refraining from static rehabilitation programs (which might be feasible in the inpatient sector), the continuous adaptation to patients' needs and context ensures the patients' empowerment to adequately deal with their circumstances. The system focuses on the change of people's attitudes and behaviors toward a healthier lifestyle and supports the elderlies in their everyday rehabilitation activities and supports better therapy adherence. In general, there would be two options in case a patient leaves the desired safe thresholds (see Figure 4 for an overview). First, the physician could intervene and take measures to come back on track. Admittedly, the physician has a great expertise and abilities to do so but is not at the patient's site and examines him/her only occasionally (given the case of home rehabilitation). Second, the VC could intervene as it is in the immediate proximity and permanently examines the patient. The VC's limited expertise has to be compensated by means of providing medical knowledge, sequence plans, or interaction schemes in terms of modes that are usable for the VC. This relates to clinical pathways (such as the representation of the adequate treatment procedure), smart-home sensors (replacing the eyes of a human caregiver) or sensors for vital data, and the avatar itself (replacing the caregiver's face and interaction possibility) or the machine learning and reasoning (replacing the human's assessment abilities).

The technical concept of vCare ties up to the preliminary contributions of former European initiatives like universAAL (ReAAL)^11^ and FI-WARE^12^, and Miraculous Life^13^, DoReMi^14^, or eWall (19) by reusing and building upon this technical groundwork.

### vCare Architecture

**Figure 5:**
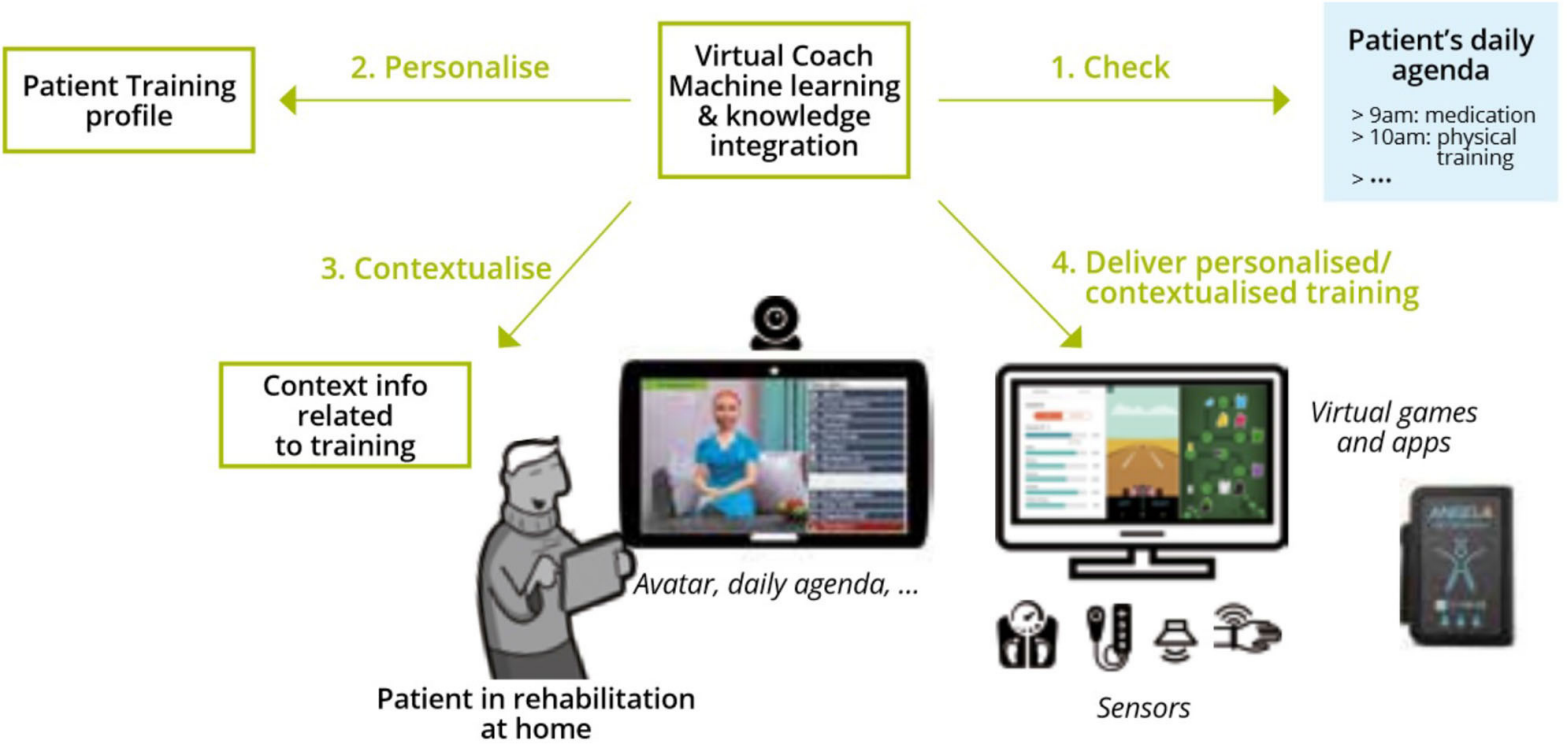
vCare main building blocks.

**Figure 6:**
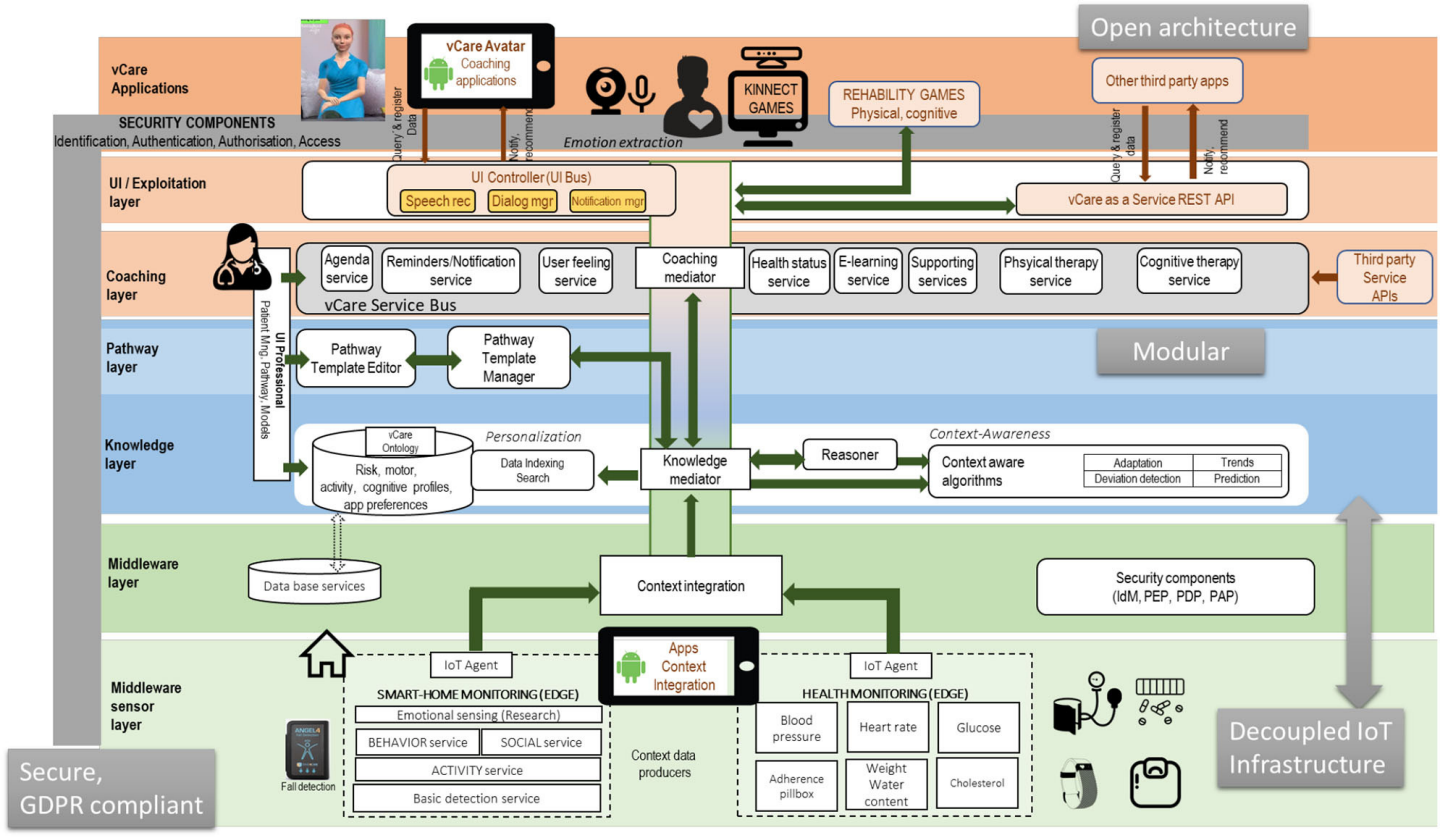
vCare system layered architecture.

vCare's architecture follows an open and decoupled design to leverage the existing solutions. vCare addresses the challenge to assemble the different existing solutions to integrate required functionalities for context integration, usage of clinical pathways, monitoring of health data, and provision of smart avatar as well as a set of serious games (see Figure 5). The design of the technical architecture is a multi-layered and service-oriented architecture (SOA). The architecture consists of a middleware sensor layer, a knowledge and pathway layer, a coaching layer, and user interface (UI) layer (see Figure 6).

The core layer is the knowledge layer, which deals with the ontology-based storage, and reasoner for personalization of the user interaction concerning the behavior of the patient and his/her care plan. This also allows data exploitation and the dynamic generation of new interactions to fulfill specific needs according to the patient profile and the specific context.

The sensor layer provides the services to integrate sensor data that provide vital data as well as context information such as movement patterns. Therefore, this sensor layer integrates open IoT platforms. vCare deployed FIWARE as the reference platform for the middleware. FIWARE has shown the greatest adoption in the market in the last years as well as covering very well cross-domain scenarios. However, for covering the specific domain of the smart-home at the edge, universAAL^11^ is an important technology to address the context semantic integration in the edge.

The highest layers in the architecture in Figure 6 take advantage of the access and enrichment because of the work in the middleware and the knowledge layer. The pathway and coaching layers bring specific services that compose the final coaching experience aiming at tailoring a personalized and in-time plan that engages patients into the coaching experience. In order to boost the innovation in the field of the coaching applications, vCare has adopted the *vCare as a service approach* in order to give access to its valuable intelligent resources. The first use case to be implemented will be to install a serious game platform to provide both physical and cognitive training. The following figure shows an overall view of the first release of the vCare platform.

The major advancement of the vCare concept is an adaptive integration and use of several coaching, monitoring, and adaptation services according to a personalized pathway. All services, devices, and sensors can be (re-)composed and re-configured if needed. The knowledge layer's agents address the relevant medical use cases by making activity or adaptation recommendations. Moreover, the knowledge layer's ontology contains the information about the patients' needs and conditions, services, and clinical pathways. The ontology gives the input for the machine learning (ML) algorithms that analyze the compliance and progress of the patient in comparison with his/her pathway. Depending on the result, adaptations of the former pathway can be proposed.

## Methods

The primary innovation for a “virtual coach” is that it aims to support health management for secondary prevention as a home self-care service (17). Currently, secondary prevention during rehabilitation is often provided in an institutionalized manner. Abstaining from that, vCare offers home rehabilitation solutions that are able to adapt dynamically depending on the situation of the patient. The system can be configured by clinical pathways in a way that enables it to be used as a solution for different NCDs. In the following subchapters, the methodological approach for reaching the desired care-related objectives is outlined.

Overall, within vCare, it is foreseen that the prototype of the VC system, once defined and developed in a highly multidisciplinary context (with the involvement of physicians, physiotherapists, neuropsychologists, bioengineers, and ICT researchers), has to be evaluated according to an incremental approach composed of different phases with the aim of testing its functionalities, usability, and reliability.

### Overall Validation Studies' Framework

Ordinarily, rehabilitative therapy begins in the acute-care hospital after the person's overall condition has been stabilized. The main clinical ambition of vCare is to fill one of the existing gaps in chronic patients' long-life management, with an ICT system able to give a patient's tailored care pathway after hospital discharge.

Neurological and cardiovascular diseases embraced in the vCare context are especially relevant for people as they age. For sure, both these pathologic conditions are quite diverging with regard to the acute onset or their chronic course but tend to converge when speaking about their pathogenic mechanisms and therapeutic treatment. That is why, fortunately, the overall procedural structure of the rehabilitation phase is still somehow similar. However, particular rehabilitation care pathways differ at some points. The main objective following a rehabilitation plan is to regain body control. In fact, patients are supported to re-learn or regain the ability to deal with their home environment without (extensive) help. Due to the major progress in the acute treatments (e.g., thrombolysis, introduction of stroke units, acute cardiac care units, and dysphagia management), the majority of people survive acute severe events. Long-term disabilities are however still very prone to appear. vCare seeks to support the rehabilitation process by reducing the disabling conditions as much as possible.

**Figure 7:**
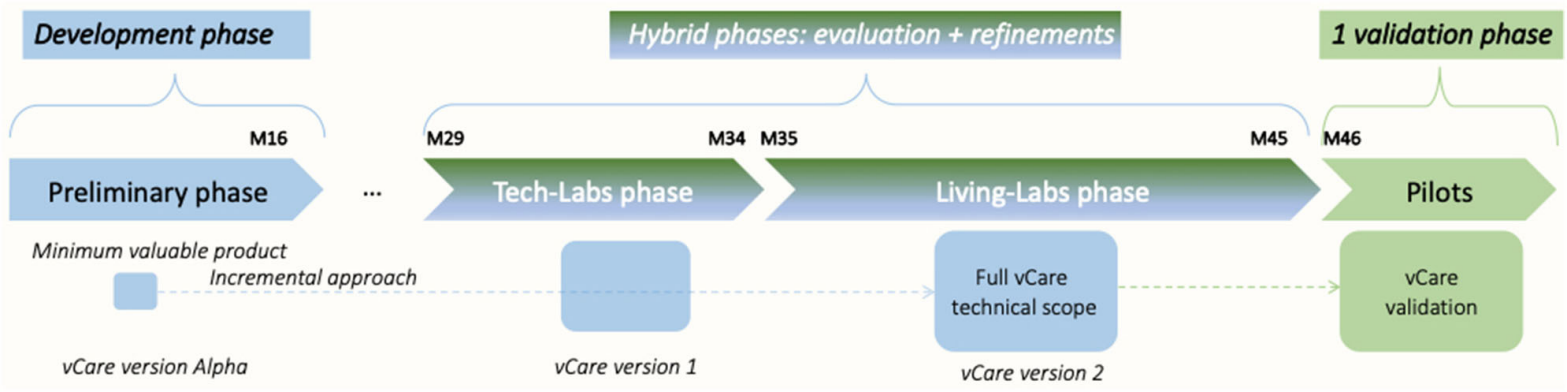
Overall implementation plan.

The different testing phases that have been identified in vCare and the overall implementation scope are depicted in Figure 7.

vCare is deploying a 2-fold joint testing phase for the validation studies. This is intended to provide real-time feedback from the patient perspective, where the patient is simulated by a physician. The users are members of the rehabilitation team, and they have to test the platform from the clinical point of view. First, the testing phases are carried out in a tech lab strictly in contact with developers. The different functional parts and services that build the entire prototype will be deployed in this laboratory site, trying the integration with external systems. The system will be refined, taking into account the results of these tests. The purpose of the pre-evaluation is to verify the compliance of the prototype to the clinical requirements or the external validity in terms of fitness with the needs of the reference sites. The vCare project has set up a tech-lab phase in three different sites (Austria, Germany, and Spain), to work in the technical integration and validation that should release the first version of the vCare service.

Second, the clinical evaluation phase will initially be performed in a controlled environment (living lab) where the patients behave like being at home while they stay in the clinic. For this, a domestic environment is usually foreseen in a rehabilitation hospital for occupational therapy purposes, equipped with project technologies. The users are real patients. Initially, a member of the rehab team supervises them. Later, they are left alone with the VC but always in a “protected” environment. In the second stage, which will be performed with a subgroup of patients, users will act alone in their home setting (real-life evaluation). To assess the compliance of the VC-supported rehabilitation scheme, the evaluation will be done based on concepts derived from clinical good practice and overall experiences together with standard procedures for rehabilitation.

Finally, the validation study (pilot test study) will evaluate the efficiency of a real home-based rehabilitation program using the VC of vCare. In this pilot study, patients will be recruited and engaged in a personalized home rehabilitation program, based on the clinical profile and the clinical status of the patients. The study will be conducted in a home setting and will be configured as a randomized controlled trial (RCT), with the enrolment of patients who will undergo home rehabilitation with the support of the VC and patients who will receive conventional rehabilitation (i.e., clinical recommendations at discharge). The overall aim of the pilot study is to refine and test the use of the vCare coaching system to find out if it is useful in motivating and empowering patients with various functional impairments and comorbidities to engage actively in performing personalized rehabilitation activities in order to be independent and to improve their QoL.

Summing up, the first step is the validation from a technical point of view without the involvement of patients. This is to primarily test the correct functioning, the infrastructure, and security of the solution (tech-lab phase). This phase is managed at the technical development centers with the supervision and the participation of clinical staff. On that basis, the living-lab phase will start. This phase aims to evaluate the usability and liking of patients and the abilities of the system of adapting according to patients' characteristics and needs in a controlled environment (special test rooms equipped by the clinical partners initially involving real patients). Third, the testing process of the vCare virtual coaching system will end with a pilot RCT (pilot test phase) that will be carried out in patients' real homes. This last and (from the clinical point of view) most important phase of “clinical validation” will be presented and is in focus in the remaining manuscript.

### Pathologies' Scenarios

The system will be evaluated for neurology's [patients affected by stroke (SD) and PD] and cardiovascular patients' rehabilitation (patients affected by HF and IHD).

The situation for these four pathologies slightly differs with regard to eligibility criteria outcome measures and care scenarios at all. Therefore, we first briefly outline background information on these four cases and the envisioned advancements (compared with the situation without the vCare solution showing how the VC can advance within the different rehabilitation scenarios). Afterwards, we continue to describe the foreseen shape and constraints of the study and how to operationalize and test the expected advancements.

#### The Stroke Scenario (Pilot Site: Milan/Italy)

##### Background

Stroke is the leading cause of disability worldwide. It causes different impairments such as loss of muscle tone and strength, sensation and coordination deficits and gait and balance disorders, motor disabilities of the upper limbs and hands, deficits in haptic, praxias, and bimanual and/or hand–eye coordination disturbances. Impairments and the related disabilities may have a significant impact on an individual's independence, safety, and QoL. Cognitive impairment can be associated with stroke and has important repercussions in everyday life as well. While stroke patients highly benefit from intensive rehabilitation program in the inpatient setting, they suffer from the lack of continuity and support of a comprehensive rehabilitation program at home to optimize motor and cognitive recovery.

##### Envisioned advancement

The home rehabilitation pathway lacks clear clinical guidelines and requires major personalization effort with regard to the patient's condition, needs, and abilities. vCare will allow to fill the gap of the care continuum between discharge from the hospital. It supports professional care and provides a reliable tool for motor and behavioral engagement supporting the patient's long-term recovery from stroke's impairments.

#### The Parkinson Disease Scenario (Pilot Site: Bilbao/Spain)

##### Background

PD is the second most common neurodegenerative disease after Alzheimer's disease. It is estimated that the prevalence is 2–3% of patients over 65 years of age. The main problems of the disease are in the motor domain (tremor, rigidity, bradykinesia, instability, and falls) and in the neuropsychiatric sphere (cognitive impairment, dementia, depression, impulsiveness, and psychosis). Most of these aspects can benefit from an intense rehabilitation program for both motor and cognitive recovery. In addition to the presence of the symptoms with the evolution time, it can be observed that these symptoms have both motor and non-motor fluctuations. This requires individualized adaptation of PD-specific rehabilitation programs (currently not available in the EU), ensuring good adherence, the same as for the pharmacological treatment, and avoiding the risks inherent to this symptomatology.

##### Envisioned advancement

There is still a lack in a comprehensive and standardized approach in implementing home-based rehabilitation. The vCare solution integrates sensors that collect information from motor and non-motor symptoms by adapting this information to specific processes of rehabilitation at home and, respectively, supporting the patients in daily life given the personal restriction caused by PD.

#### Heart Failure (Pilot Site: Bucharest/Romania)

##### Background

For HF patients, cardiac rehabilitation represents a professional program, medically supervised, and individualized accordingly to the disease and to the needs of every patient. It has a decisive role in helping patients recover after an unwanted cardiovascular event and mainly to help in getting healthier and in passing easily through the early outpatient period. This includes educational services and guidance meant to support the patients in changing their lifestyle, to improve the effort capacity and the overall health, and to reduce the risk of future cardiac events.

##### Envisioned advancement

It is important to offer a different intelligent model of home rehabilitation based on rules and machine learning supported by a VC, which could improve uptake in cardiac rehabilitation programs and ensure longevity by tackling some of the mentioned barriers (e.g., availability of the medical personnel to monitor and adjust patient's rehabilitation plans remotely).

#### Ischemic Heart Disease (Pilot Site: Aarhus/Denmark)

##### Background

IHD is one of the leading causes of death worldwide (20). However, mortality rates have been falling over the last two decades, partly explained by improvements in treatment and primary prevention (21). Therefore, cardiac rehabilitation and patient education are essential to support these people to manage their symptoms and risk factor and lifestyle control to improve their prognosis and support them to enhance coping strategies to live with the disease and recover from a possible functional loss (22). Cardiac rehabilitation with patient education is evidenced as a safe intervention to improve health-related QoL, affect the risk factor and lifestyle profile in a positive direction, and reduce cardiovascular mortality and readmissions (23). However, approximately half of the patients with IHD have difficulties to achieve and maintain risk factor reductions and beneficial lifestyle changes during the outpatient period (24–26). Therefore, there is a need to offer the people with IHD a continuous cardiac rehabilitation, which goes beyond the mandatory cardiac rehabilitation and which can take place in the person's home environment.

##### Envisioned advancement

For IHD patients, it is important to offer a transition to cardiac rehabilitation in the home setting after completing phase II, as there is a substantial lack of uptake and lack of adherence to both the phases. A VC system interacting and coaching with the patients in their home might be a solution to manage risk and lifestyle control and to improve QoL.

### Study Design and Sample Size

The pilot study is designed as a prospective interventional study whose primary purpose is supportive care. The study will be conducted in a home setting and will be configured as an RCT. Specifically, the physician will recruit patients upon discharge from the hospital, following inclusion and exclusion criteria and will plan a personalized rehabilitation program for each subject. For each site, a subset of 10 patients (for each disease) will follow a rehabilitation program at home with vCare for a 6-month period. A second group of 10 patients (for each disease), enrolled as a control group and matching demographic characteristics, will be used for the comparison in terms of clinical and economic efficiency.

Only for HF patients will a third group be enrolled: it will consist of patients who will have to perform 6 months' ambulatory rehabilitation program which consists of three 45-min physical activity sessions per week, one monthly psychological session, and one monthly dietary session. Following the 6 months' period, all three groups will perform a cardiovascular evaluation to assess their evolution.

#### Recruitment and Eligibility Criteria

Each of the four clinical centers will recruit 10 patients (for a total of 40 patients) to use the VC system individually at home.

##### Stroke

Twenty patients affected by SD conditioning a physical and/or cognitive long-term disability and discharged home after an intensive inpatient rehabilitation program will be recruited.

Inclusion criteria: (i) patients who suffered a recent (<1 month) stroke (either ischemic or hemorrhagic) confirmed by CT and/or MRI scan, with a National Institutes of Health (NIH) Stroke Scale/Score (NIHSS) score of ≤ 14, corresponding to a mild–moderate neurological impairment; and (ii) age ≥ 65 years.

Exclusion criteria: (i) global aphasia; (ii) presence of cognitive impairment [i.e., Mini-Mental State Examination (MMSE) ≤ 22]; (iii) any evidence of a serious condition that may affect participation in the study (e.g., traumatic brain injury and oncological, hepatic/renal, immune-based, metabolic/endocrine, psychiatric, respiratory, or infective disorders); and (iv) unable to understand and comply with the protocol or to give informed consent.

##### Parkinson's disease

Twenty patients with PD, in a stage that causes them to have a disability with motor and non-motor symptoms (mainly cognitive), will be recruited. After a rehabilitation period at the hospital, adapted to their stage of illness, they are discharged to follow a personalized rehabilitation program.

Inclusion criteria: (i) patients diagnosed with PD according to established clinical criteria (Brain Bank of London); (ii) age > 65 years; (iii) patients with scores higher than 60% on the daily life activities scale of Schwab and England; (iv) presence of motor fluctuations perceived by the patient; and (v) Hoehn and Yahr stage 1 and 4.

Exclusion criteria: (i) patients diagnosed with atypical parkinsonism; (ii) patients with scores below 24 on the Montreal Cognitive Assessment (MOCA) scale; (iii) specifically exclude bedridden or dependent patients; (iv) patients with other chronic diseases such as HF, severe lung, or liver problems; (v) patients with severe psychiatric problems such as hallucinations or major depression; (vi) patients with poor adherence prior to pharmacological or rehabilitative treatment; and (vii) unable to understand and comply with protocol and/or give informed consent.

##### Heart failure

Thirty patients who suffered an HF hospitalization that will match the inclusion criteria and will not have any of the exclusion criteria will be enrolled.

Inclusion criteria: (i) diagnosis of HF class New York Heart Association (NYHA) II–III; (ii) age > 65 years; and (iii) hospitalized in the cardiology department of the Romanian clinical reference site.

Exclusion criteria: (i) unstable angina; (ii) systolic resting blood pressure > 200 mmHg, diastolic blood pressure > 110 mmHg; (iii) blood pressure decrease with more than 20 mmHg at standing position; (iv) severe aortic stenosis; (v) sepsis; (vi) uncontrolled arrhythmias; (vii) uncontrolled atrial tachycardia heart rate (HR) > 120 bpm; (viii) decompensated HF; (ix) atrial-ventricular grade 3 transmission block; (x) recent pulmonary thromboembolism; (xi) phlebitis; (xii) persistent ST segment elevation of more than 2 mm; (xiii) uncontrolled diabetes; (xiv) locomotor disabilities; (xv) thyroiditis, hypokalemia, hyperkalemia, and hypervolemia; and (xvi) unable to understand and comply with protocol and/or give informed consent.

##### Ischemic heart disease

Twenty patients affected by IHD, discharged from hospital and having finished a 12-week hospital-controlled mandatory cardiac rehabilitation program, will be recruited.

Inclusion criteria: (i) patients hospitalized with IHD, either an acute ST- or non-ST myocardial infarction or a condition with unstable or stable angina pectoris requiring a percutaneous coronary intervention, a coronary artery bypass grafting, and/or medical treatments; (ii) age ≥ 65 years; and (iii) finished with the 12-week hospital-controlled mandatory cardiac rehabilitation program with permission from cardiologist to transit to phase III cardiac rehabilitation.

Exclusion criteria: (i) unstable condition requiring treatment; (ii) unstable angina pectoris; (iii) uncontrolled sustained arrhythmias; (iv) severe valve diseases; (v) severe uncontrolled hypertension with resting systolic blood pressure > 180 and diastolic blood pressure > 110 mmHg; (vi) active myo-, peri-, or endocarditis; (vii) recent pulmonary embolism; and (viii) unable to understand and comply with protocol and/or give informed consent.

### Objectives of the Pilot Study

The overall aim of the pilot study is to test and refine the use of the vCare coaching system so that it can motivate and empower patients with various functional impairments and comorbidities to engage actively in performing personalized rehabilitation activities, improving their independence and QoL.

The specific objectives of the pilot study are defined as follows: (i) enhancement of QoL (primary outcome); (ii) risk factor reduction; (iii) better adherence to care plan at home; and (iv) personalization and health promotion. This is operationalized for the four relevant pathologies in the following.

Furthermore, the pilot study will also test usefulness, feasibility, and effectiveness of virtual coaching in home setting in neurological and cardiologic patients.

#### Primary Outcome Measure Information

##### Stroke

Enhancement of QoL Measure, in Particular Expecting

1. increase of the QoL score (SF-12), at the end of in-house pilot study period (6 months),
2. increase of the SF-12 mental sub-score (Mental Component Summary),
3. increase of the QoL score on the Stroke Self-efficacy Questionnaire, at the end of in-house pilot study period (6 months).

The questionnaires will be administered to the two groups of subjects at the beginning of the study and after a follow-up of 6 months. A trained health professional will administer the questionnaires.

##### Parkinson's disease

Enhancement of QoL measure, in particular expecting

1. increase of the QoL score (SF-12), at the end of in-house pilot study period (6 months),
2. 15% increase of the SF-12 mental sub-score (Mental Component Summary),
3. 10% increase of the QoL score (PDQ-39), at the end of in-house pilot study period (6 months).

SF-12 and PDQ-39 questionnaires will be administered to the two groups of subjects at the beginning of the study and after a follow-up of 6 months. A trained health professional will administer the questionnaires.

##### Heart failure

Enhancement of QoL measure through SF-12, in particular expecting

1. increase of the QoL score (SF-12), at the end of in-house pilot study period (6 months),
2. increase in the Minnesota Living with Heart Failure Questionnaire (MLHFQ) score.

SF-12 and MLHFQ questionnaires will be administered to the three groups of subjects at the beginning of the study and after a follow-up of 6 months. A trained health professional will administer the questionnaires. Increased cardiovascular fitness will be defined as increase with more that 10% of the exercise capacity measure in the VO2max (maximal oxygen consumption by the body measured in ml/kg/min).

##### Ischemic heart disease

Enhancement of QoL measure through SF-12, in particular expecting

1. 10% increase of the QoL score (SF-12), at the end of in-house pilot study period (6 months),
2. 15% increase of the SF-12 mental sub-score (Mental Component Summary).

SF-12 questionnaires will be administered to the two groups of subjects at the beginning of the study and after a follow-up of 6 months. A trained health professional will administer the questionnaires.

#### Secondary Outcome Measure Information

The secondary outcome measures will emerge from the vCare platform, as follows.

##### Risk factor reduction

Risk factors are disease related, and their detection depends on the rehabilitation pathway. While piloting vCare, the patient's lifestyle will be monitored, and specific risk factors will be determined. The goal is to reduce each specific risk factor by at least 10% of the baseline value at the end of the study.

##### Stroke

The primary risk factor for the subjects with stroke will be related to the considered tendency toward inactivity, measured according to the following parameters:

1. daily number of steps, expected to improve at least by 10% in 6 months;
2. time spent exercising, expected to improve at least by 10% in 6 months.

Rate of falls and fall risk reduction will be first investigated in the living lab, and if feasible, they will then be measured in the home setting also, as monthly number of falls. In the behavioral context, the tendency toward a less risky and more correct behavior will be measured according to the weekly number of corrective e-learning lessons that followed.

##### Parkinson's disease

The primary risk factor in patients with PD will be the increase in OFF time and detection of specific gait problems such as freezing, measured through the following parameters.

1. OFF time in hours, expected to improve at least by 10% in 6 months;
2. daily number of steps, expected to improve at least by 10% in 6 months;
3. number of freezing episodes of the march, expected to improve at least by 10% in 6 months.

##### Heart failure

The primary risk factor for the subjects with HF will be related to sedentary lifestyle, measured according to the following parameters:

1. daily number of steps, expected to improve at least by 10% in 6 months;
2. time spent exercising, expected to improve at least by 10% in 6 months;
3. more than 15% reduction in low-density lipoprotein (LDL) cholesterol levels.

In the behavioral context, the tendency toward a less risky and more correct behavior will be measured according to the weekly number of corrective e-learning lessons followed. Moreover, control of vital parameters will be taken into consideration (Table 11).

##### Ischemic heart disease

The risk factors monitored for the subjects with IHD according to the Use Cases will be related to sedentary lifestyle, measured according to the following parameters:

1. daily number of steps, expected to improve at least by 10% in 6 months;
2. time spent exercising, expected to improve at least by 10% in 6 months.

Also changes in weight loss (improved physical activity), medication adherence, anxiety reduction, and smoking cessation will be taken into consideration. In the behavioral context, the tendency toward a less risky and more correct behavior will be measured according to the weekly number of corrective e-learning lesson that followed.

##### Better adherence to care plan at home

1. Total no. of subjects that adhere to the Care Plan with *vCare*, raised up to 80%.
  Treatment adherence will be evaluated by monitoring access to the vCare platform and/or interaction with the VC at least once a week for a continuative period of 3 months.
  These actions include turning on the tablet and/or smart TV to access the platform, receiving feedbacks from the VC to the patient and vice versa, performing of at least one suggested activity per week, and recording of vital parameters. In other words, through the data collected inside the platform, the compliance of the patients will be assessed, expecting 80% of the subjects to engage consistently with the home program.
2. No. of rehabilitation treatments, performed autonomously by the patient supported by *vCare*, raised up to 70%.

It is expected that 70% of the rehabilitation plan, as in 70% of the proposed activities by the VC, should be followed, on average in 3 months.

##### Personalization and health promotion

1. Refinement rate of rehabilitation therapy (exercises and programs) raised up to the 60% of the cases that require a change of treatment.

The refinement rates have been hypothesized as the number of changes in the pathway solutions suggested by the vCare solution during the pilot test. The result shows the times in which the pathway changes.

In order to measure acceptability and identify the characteristics of subjects who were likely to reject technological health services, the SUTAQ (Service User Technology Acceptability Questionnaire) will be administered at 6 months from the pilot test start, expecting to score in the highest quartile.

## Anticipated Results

The overall aim of the application of the vCare's VC is to improve the patient's QoL after an acute episode or chronic disease-related rehabilitation. The vCare system will be a persuasive and non-obtrusive technology intended to help people change their habits and improve their home-based rehabilitation. Therefore, they can become healthier and gain more healthy time of living. The expected results are grouped into the following categories, based on four clinical objectives: Recovery of an Active and Independent Life at Home; Risk Factor Reduction; Better Adherence to Care Plan; and Personalization and Health Promotion (Figure 1). Here, we give an overview and brief discussion of the study objectives to be achieved as already discussed in *Objectives of the Pilot Study*.

### Recovery of an Active and Independent Life at Home

The approach of rehabilitation guidance by a virtual coaching system does not intend to replace specialists but rather complement their work to guarantee the continuity of care in the home environment and daily life where current rehabilitation programs often fail because of the lack of continuous assistance. The VC should lead to a continuous reduction of risk factors that are related to the probability of a relapse of the disease, the manifestation of disabilities, or the decline of mental health.

The global value of a rehabilitation program for a patient is hard to be evaluated in an objective manner because it is related to the individual's satisfaction or happiness with life in domains that he or she considers as important. The results of rehabilitation are often evaluated by applying standardized clinical scales that evaluate physical improvements and/or functional impairments, but these scales omit the measures of the patient's own perception of the mental and emotional effects of the bodily disabilities. Because of this, the state-of-the-art of QoL evaluation is based on predefined standardized tests like the SF-12. Taking into consideration the way in which patients view their own health situation is the most important element of patient-centered health care. Given the complex nature of clinical evaluation procedures, evidence regarding effects between QoL, and patient engagement is quite weak. Thus, vCare aims to enhance the QoL and provide a reliable basis with a 10% increase of the QoL score (SF-12), as off the in-house pilot study durations (6 months) and a 15% increase of the SF-12 mental sub-score (Mental Component Summary).

### Risk Factor Reduction

A relapse or decline of health status closely depends on the evolution the patients' risk factors (e.g., blood pressure, weight, physical and cognitive activity, and social life). The rehabilitation supported by a VC should facilitate the reduction of risk factors. The risk factors are disease related, and their detection depends on rehabilitation pathway (i.e., post-acute and chronic). While piloting vCare, the patient's lifestyle will be monitored, and specific risk factors (i.e., physical activity and falls risk) will be determined. The goal is to reduce each specific risk factor of at least 10% of the baseline value, at discharge.

### Better Adherence to Care Plan

Often, patients disregard discharge recommendations or prescribed therapy protocols. The rates of non-adherence to chronic illness regimens are estimated to be between 30 and 60% (27), which is significantly worse than those for acute illness treatments. Low adherence to medical recommendations takes many forms. People may miss visits, do not cope with a recommended treatment, do not complete recommended behaviors (e.g., exercise changes), adjust their medication plans by themselves, or prematurely discontinue therapy. The virtual coaching solution should support the adherence to care plan by providing constant assistance to follow the foreseen treatment pathway. Therefore, the VC becomes an essential supplement to the direct contact to the specialists. vCare expects that the number of subjects that adhere to the care plan with vCare will be raised up to 80%, while the number of rehabilitation treatments, performed autonomously by the patient supported by vCare, raised up to 70%.

### Personalization and Health Promotion

Home rehabilitation eases the alignment of the rehabilitation process with the patients' daily activities in his/her her home surroundings. To align the rehabilitation program to the physical, cognitive, and social status of the patient, it has to be personalized to patients' needs and conditions. Especially when it comes to non-chronic diseases, adequate health promotion can support long-term behavioral changes, decreasing the economic costs and the probability of a relapse. vCare expects a refinement rate of rehabilitation therapy (exercises and programs) raised up to the 60% of the cases where a change of treatment is required. The acceptance of the VC (by professionals and patients) will be assessed through specific questionnaires (e.g., System Usability Scale), measured in the highest quartile score (28).

## Discussion

Rehabilitation after an acute episode or in a chronic disease is a key aspect for patients' management and is a main contributor to their increase in the QoL associated with improved self-care. Overall, inpatient multidisciplinary rehabilitation remains one of the cornerstones in the treatment of disabilities. Rehabilitation strategies particularly support patients in relearning skills that are lost and adapting healthy habits. However, the lack of continuity of care after the inpatient care and rehabilitation leaves a gap in the continuum of the fragile patients' management (having a need for a constant, well-structured rehabilitation process). The patients' motivation should be sustained during physical activity sessions in order to maximize the rehabilitation outcome. Motor actions need to be practicable in different tasks and environmental contexts to develop motor schemata that are versatile enough to meet the situations they encounter in daily life. Therefore, coaching systems must activate appropriate strategies to improve the motor and cognitive impairments of patients. vCare's VC will continuously apply and provide personalized rehabilitation recommendations, so that patients with such impairments will be supported and coached to improve their body functions and healthy habits. By doing so, the factors limiting the patients' reinsertion into their home or community and living independently will be diminished. In the digital era that we live in, the technological advances should provide a solid ground for developing new rehabilitation practices that could help patients with NCDs to improve their health condition. vCare will combine at the same time with technological advancements in terms of sensors devices for monitoring the patient's vital parameters and location with the advantages provided by the gamification elements in terms of therapeutic serious games, pseudo-human interaction possibilities by means of the virtual avatar, and machine learning abilities. All these will be supporting the personalization and adaptation of the treatment based on the initial plan, set rules, acquired data, and inferred behavioral patterns, blended with insights from clinical pathways.

The concept of vCare is currently under validation first in tech labs at the present time, aiming to provide clear evidence about its impact on the rehabilitation process with regard to the efficiency and the tangible outcomes to the QoL, independent living, recovery, risk factor reduction, better adherence, and personalized health promotion of patients, as described in *Anticipated Results*. Limitations, such as low adoption level, cannot be excluded; however, they have been identified as risks with a mitigation strategy. Furthermore, the ethical approval process at the clinic reference sites' Ethical Committees has started, and approvals are confirmed. This is the basis to start the test phases with the patients in Autumn 2020.

## Data Availability Statement

The original contributions presented in the study are included in the article/supplementary material, further inquiries can be directed to the corresponding author/s.

## Author's Note

The virtual coach solution makes it possible to utilize a means of communication for the patient. Acting like a physical coach, the VC channels the information and provides suitable recommendations to the patient, as well as engages the patient “like a coach” to stay in their “training plan” and also provides a backlink of the outcomes as well as a possibility of intervention to the physician. This paper summarizes the core concepts and design decisions of the European vCare project.

## Author Contributions

All authors listed have made a substantial, direct and intellectual contribution to the work, and approved it for publication.

## Conflict of Interest

JR and AM were employed by MYSPHERA. LP was employed by Imaginary s.r.l. The remaining authors declare that the research was conducted in the absence of any commercial or financial relationships that could be construed as a potential conflict of interest.

## Footnotes

^1^WHO. *Noncommunicable Diseases Progress Monitor*. Vol. 46, Geneva: World Health Organization (2017).

^2^*Chapter 4:‘Rehabilitation’ In: World Report on Disability*. Available online at: http://www.asksource.info/resources/chapter-4-rehabilitation-world-report-disability-p93-134.

^3^*Exercise Rehabilitation - An Overview | ScienceDirect Topics*. Available online at: https://www.sciencedirect.com/topics/medicine-and-dentistry/exercise-rehabilitation.

^4^*HOLOBALANCE Project*. Available online at: https://holobalance.eu/.

^5^*EMPATHIC Project*. Available online at: http://www.empathic-project.eu/.

^6^*Council of Coaches Project*. Available online at: http://council-of-coaches.eu/.

^7^*NESTORE Project*. Available online at: https://nestore-coach.eu/home.

^8^*CAPTAIN Project*. Available online at: https://www.captain-eu.org/.

^9^*AHA Project*. Available online at: https://ec.europa.eu/eip/ageing/home_en.

^10^*I2M Project*. Available online at: https://ec.europa.eu/eip/ageing/innovation-market-i2m_en.

^11^*universAAL*. Available online at: https://www.universaal.info/.

^12^*FIWARE*. Available online at: https://www.fiware.org/.

^13^*Miraculous Life*. Available online at: http://www.miraculous-life.eu/.

^14^*DOREMI*. Available online at: http://www.doremi-fp7.eu/.
